# UNDERSTANDING POTENTIAL INVOLVEMENT OF CARE PARTNERS IN CAPABLE

**DOI:** 10.1093/geroni/igac059.1297

**Published:** 2022-12-20

**Authors:** Beth Fields, Pamela Toto

**Affiliations:** University of Wisconsin-Madison, Madison, Wisconsin, United States; University of Pittsburgh, Pittsburgh, Pennsylvania, United States

## Abstract

This qualitative study focused on understanding how to involve unpaid care partners (‘family members or friends’) of older adults who are participating in CAPABLE. Using field notes, focus groups, and key informant interviews, the study revealed considerations for involving care partners, including providing choices, defining roles, sharing information in a collaborative manner, and reinforcing knowledge and skills training. These findings can help guide organizations interested in involving care partners in their older adult programs.

